# *BRAF V600* mutation profiling in primary skin nodular melanoma in Indonesia: an analysis using high resolution pyrosequencing

**DOI:** 10.1186/s13104-020-05000-w

**Published:** 2020-03-18

**Authors:** Hanggoro Tri Rinonce, Rovi Panji Mustiko Aji, Ni’mah Hayati, Maria Fransiska Pudjohartono, Bidari Kameswari, Sumadi Lukman Anwar, Irianiwati

**Affiliations:** 1 Department of Anatomical Pathology, Faculty of Medicine, Public Health, and Nursing, Universitas Gadjah Mada/Dr. Sardjito Hospital, Sleman, Yogyakarta, 55281 Indonesia; 2 Department of Anatomical Pathology, dr. Soeradji Tirtonegoro Hospital, Klaten, Central Java 57424 Indonesia; 3 Department of Surgery, Faculty of Medicine, Public Health, and Nursing, Universitas Gadjah Mada/Dr. Sardjito Hospital, Sleman, Yogyakarta, 55281 Indonesia

**Keywords:** Melanoma, Skin cancer, BRAF, Pyrosequencing, Indonesia

## Abstract

**Objective:**

We aimed to investigate the prevalence and type of *BRAF V600* mutations and the associations with clinicopathological variables in primary skin nodular melanoma cases in Yogyakarta and Central Java, Indonesia using pyrosequencing.

**Results:**

*BRAF V600* mutations of the V600E type were found in 21 (53.85%) specimens. The variant allele frequencies (VAFs) ranged from 5.07 to 94.70%, averaging 29.05%. However, most cases had low VAFs, with 13 (61.9%) specimens below 20% and 4 (19.05%) below 10%.

## Introduction

Despite its low incidence, melanoma remains the deadliest skin malignancy and the main contributor to deaths from skin malignancies. With a global death toll of 60,712 deaths in 2018, this disease is responsible for over half the mortality from skin cancer [1]. The dismal outcomes for melanoma patients call for further research to improve their management, which is currently focused on molecular profiling of melanomas.

The *BRAF V600* is the most commonly found somatic mutation in melanomas and thus has become a potential marker and therapeutic target [2]. There is a wide range of research on this mutation, from its role in melanoma pathogenesis to the development of specific targeted therapy. Even nowadays, application of the *BRAF V600* mutation for diagnostic and prognostic purposes is still under investigation. However, not all countries are equally represented in these bodies of data. For example, there is a paucity of studies from Asian and African countries, in which patterns of clinical behavior and underlying molecular pathogenesis are relatively different from Caucasians.

The under-representation of Asian populations is an important issue to address in melanoma research. Asia accounts for 18.6% of deaths caused by melanomas, despite only providing 7.6% of new cases worldwide [3]. Asian melanoma cases differ in characteristics from Caucasians, such as predominance of the acral lentiginous subtype and low BRAF mutation prevalence. Variations also exist within certain parts of Asia and even between different ethnicities [4, 5].

In Indonesia, melanoma cases are relatively rare but lethal, with only 1.392 new cases but 797 deaths per year [6]. Indonesian melanoma cases have distinct clinical features, showing different clinical presentations and histopathological subtypes from other Asian populations. As opposed to the acral lentiginous melanoma subtype predominance in Asia, most cases from Indonesia are of the nodular subtype, which is associated with worse prognosis [7]. The prevalence of *BRAF V600* mutations in melanoma patients from Indonesia has also been shown to be relatively low. In our previous study, we investigated the prevalence of the *BRAF V600* mutation among Indonesian melanoma cases using real-time polymerase chain reaction (RT-PCR) as the detection method and obtained a low percentage compared to Asia and other countries [8]. To our knowledge, previous researches in Indonesian populations have only used immunohistochemistry or RT-PCR methods in their studies [8, 9]. With clinical consideration that BRAF inhibitors are indicated for all *BRAF V600* mutation-positive tumors, the detection of BRAF mutations must be done with more sensitive testing methods.

Therefore, we aimed to investigate the prevalence and type of *BRAF V600* mutations using pyrosequencing in primary skin nodular melanoma in Yogyakarta and Central Java, Indonesia. With the higher sensitivity of this method, more patients with BRAF mutations might benefit from targeted therapy.

## Main text

### Materials and methods

This retrospective cross-sectional study was done at Dr. Sardjito Hospital and dr. Soeradji Tirtonegoro Hospital which were the main referral hospitals in Yogyakarta Province and Central Java Province, Indonesia. Paraffin-embedded tissue specimens from primary skin nodular melanoma cases in 2011–2018 were used as samples. Thirty-nine specimens from Javanese patients were included in analysis. DNA extraction from formalin-fixed paraffin-embedded (FFPE) primary skin nodular melanoma tissue was performed after selection of tumor-rich slides using the GeneAll^®^ Exgene^TM^ DNA Extraction Kit (GeneAll Biotechnology, Seoul, Korea) according to the protocol provided by the producer.

We assessed *BRAF V600* mutation status using pyrosequencing. Each 25 ng sample of DNA was amplified using 1× PCR buffer (Invitrogen, Frankfurt, Germany), 1.5 mM MgCl_2_, 200 μM dNTPs, 0.5 U HotStart *Taq*-Polymerase (Platinum Taq™ DNA polymerase, Invitrogen, Frankfurt, Germany), and a 1:9 mixture of forward (5′-CCTAAACTCTTCATAATGCTTGC-3′) and reverse (5′-GGGACACCGCTGATCGTTTAAACTCAG-3′) primers with universal biotinylated primers (5′-GGGACACCGCTGATCGTTTA-3′). Thermocycling was performed with preheating 95 °C for 4 min and 40 cycles of 95 °C for 15 s, 60 °C for 30 s, and 72 °C for 20 s, followed by 72 °C for 3 min using Biometra PCR Thermocycler (Analytic Jena AG, Jena, Germany). Pyrosequencing was then performed with PyroMark Gold RQ96 Reagent (QIAGEN GmbH, Hilden, Germany), 1× annealing buffer (200 mmol/L Tris acetate, 50 mmol/L Mg-acetate) after separation of biotinylated sequence using 3 μL streptavidin sepharose (GE Healthcare Life Sciences, Amersham, UK) and binding buffer (10 mmol/L tris–HCl, 2 M NaCl, 1 mmol/L EDTA, 1 ml/L Tween 20) using PyroMark Q96 Vacuum workstation (QIAGEN GmbH, Hilden, Germany). The pyrosequencing primer was 5′-TGATTTTGGTCTAGCTACAG-3′. Pyrograms and quality control of the samples were analysed using PyroMark Q96 Software ^®^ (QIAGEN GmbH, Hilden, Germany).

Clinicopathologic data were obtained from medical records. The data collected included age, sex, anatomic location, lymph node metastasis, tumor thickness, ulceration, mitotic index, necrosis, lymphovascular invasion, and tumor-infiltrating lymphocytes (TILs). The lymph node metastasis, tumor thickness, ulceration, necrosis, lymphovascular invasion, and TILs were observed from hematoxylin–eosin stained slides, while the mitotic index was calculated from Ki-67 immunohistochemistry-stained slides as previously described in our study [8]. We used Ventana Ultra Cell Conditioner 1 solution (Ventana Medical Systems, Tucson, AZ, USA) under pH 8–9 in 64 min on 95 °C for antigen retrieval and the monoclonal Ki67 antibody (Abcam, Cambridge, MA, USA) for the immunostaining according to the protocol previously described in our study [8]. The associations between *BRAF* mutation status and clinicopathologic parameters were analyzed by the Chi square test or Fisher’s exact test for categorical variables, and the independent *t*-test or Mann–Whitney test for continuous variables.

## Results

The patients’ age ranged from 21 to 80 years, with an average of 60.67 years of age. Seventeen (43.59%) patients were male and twenty-two (56.41%) patients were female. Twenty-nine patients (74.36%) had tumors on extremities, while 10 (24.64%) had tumors on the trunk or head and neck (centrally located).

**Fig. 1:**
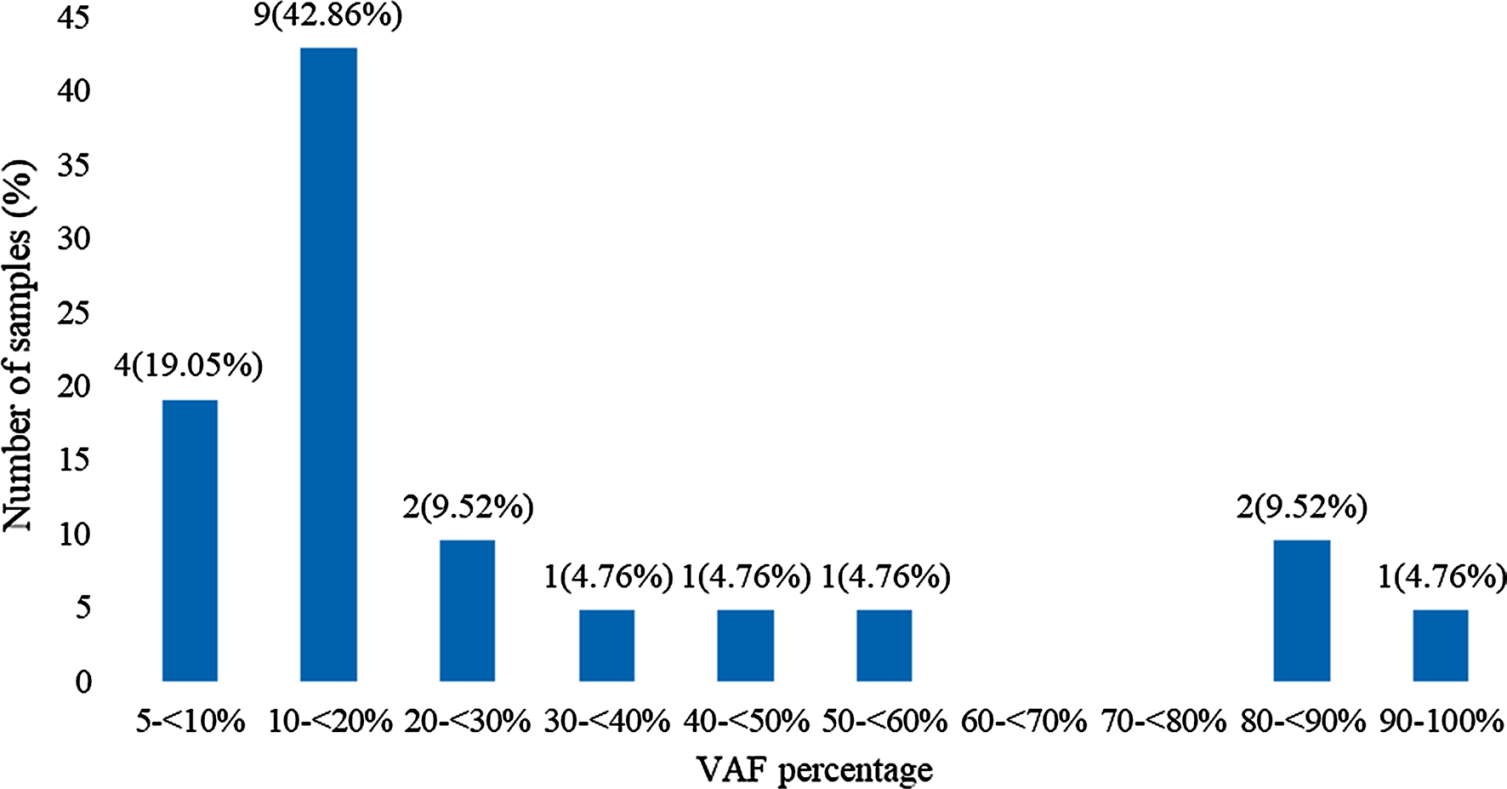
The distribution of *BRAF V600* mutation variant allele frequencies

Out of the thirty-nine samples, *BRAF V600* mutations were found in twenty-one (53.85%) samples. Variant allele frequencies (VAFs) ranged from 5.07 to 94.70%, with an average of 29.05% and standard deviation of 26.77%. Fifteen (71.4%) samples had VAFs below 30%, while 13 (61.9%) samples had VAFs below 20%. All mutations were of the V600E subtype. The complete distribution of VAFs is shown in Fig. 1. No statistically significant associations were found between the *BRAF V600* mutation status and the clinicopathological characteristics analyzed (Table 1).

**Table 1:** The association between *BRAF V600* mutation status and clinicopathologic characteristics

	BRAF (+)	BRAF (−)	*p* value^**^
Age, mean ± SD^*^	60.67 ± 15.81	60.67 ± 13.49	1.000
Age category, n (%)			
< 65 years	13 (33.33)	10 (25.64)	0.469
≥ 65 years	8 (20.51)	8 (20.51)	
Sex, n (%)			
Male	8 (20.51)	9 (23.08)	0.336
Female	13 (33.33)	9 (23.08)	
Anatomic location, n (%)			
Extremity	14 (35.90)	15 (38.46)	0.207
Central	7 (17.95)	3 (7.69)	
Lymph node metastasis, n (%)			
Present	10 (25.64)	8 (20.51)	0.549
Absent	11 (28.21)	10 (25.64)	
Tumor thickness, n (%)			
≤ 4 mm	1 (2.56)	3 (7.69)	0.246
> 4 mm	20 (51.28)	15 (38.46)	
Ulceration, n (%)			
Present	10 (25.64)	12 (30.77)	0.192
Absent	11 (28.21)	6 (15.38)	
Mitotic index, mean ± SD^*^	25.82 ± 18.77	23.22 ± 16.08	0.648
Mitotic index category, n (%)			
≥ 20%	12 (30.77)	10 (25.64)	0.588
< 20%	9 (23.08)	8 (20.51)	
Necrosis, n (%)			
Present	17 (43.59)	11 (28.21)	0.570
Absent	4 (10.26)	7 (17.95)	
Lymphovascular invasion, n (%)			
Present	9 (23.08)	4 (10.26)	0.153
Absent	12 (30.77)	14 (35.90)	
Tumor-infiltrating lymphocytes, n (%)			
Present	13 (33.33)	13 (33.33)	0.368
Absent	8 (20.51)	5 (12.82)	

*SD standard deviation

**p value < 0.05 was considered significant

## Discussion

In this study, the *BRAF V600* mutation was found in 53.85% of the nodular melanoma cases. This result resembles the mutation prevalence found in Caucasian populations, which range around 40–60% [10]. Data on the nodular subtype from Asia is scarce with two studies reporting rates of 50 and 29.4% from Japan and Turkey, respectively [11, 12]. The outcomes of our research are especially at odds with the result of our previous study using RT-PCR, which report a 10% *BRAF V600* prevalence among Indonesian nodular melanomas [8]. The higher prevalence found in this study may arise from the difference in methods. In terms of sensitivity, pyrosequencing is superior compared to high-resolution melt PCR studies [13]. In a comparation of methods, RT-PCR detected 98% of cases while pyrosequencing had 100% sensitivity [14].

Analysis of the VAF in our study shows a wide variation of 5.07 to 94.70%, with an average of 29.05%. However, most of the cases had low VAFs, with 13 (61.9%) specimens below 20% and 4 (19.05%) below 10%. For comparison, a previous study on BRAF mutation profiling in melanomas report a VAF below 20% only in 28% of samples and below 10% in 13% [15]. Given the dominance of low VAF percentages, many mutation-positive tumors which would have been detectable by pyrosequencing would likely go undetected in RT-PCR studies. The low VAF distribution in the Indonesian population in general may explain the difference in the mutation prevalence found when using RT-PCR and pyrosequencing despite the slight differences in sensitivity. The difference might be also influenced by the low sample size.

When compared to other Asian countries, the *BRAF V600* prevalence in this study is relatively high. This mutation is found in 11.9 to 41.8% of Asian melanoma cases in general [5, 12, 16, 17]. The only two studies on Asian nodular melanoma cases reported the mutation prevalence at 29.4 and 50% [11, 12]. This suggests that Indonesian melanoma cases have a high *BRAF V600* mutation prevalence, but at low intratumoral mutation rates. Variations in melanoma subtype, ethnicity, and different mutation detection methods may partially be responsible for the differences found.

No significant associations were found with clinicopathologic characteristics. The general trends, such as younger age, central location, absence of ulceration, and presence of lymphovascular invasion, mirror the findings of previous Asian studies. In our previous study done with RT-PCR in nodular melanoma patients in Indonesia [8], central location and lymphovascular invasion were correlated with *BRAF V600* mutation. The differences could have been caused by the low VAFs found in this study, which may cause mutation-positive tumor to behave like mutation-negative tumors instead. Another factor that possibly dissipated any correlations would be the unbalanced proportions of patient characteristics, as the subjects in this study were dominated by advanced extremity-located melanomas.

The presence of TILs is one of the indicators of the immune response to melanomas and is associated with better prognosis. Oncogenic BRAF mutations are associated with immune escape of tumor cells and a lower presence of TILs [18]. In our study, BRAF mutation-positive tumors tended to have a lower presence of TILs, despite not reaching statistical significance. Along with the other associations, this trend suggests that Indonesian nodular melanoma cases with the *BRAF V600* mutation tend to have worse prognoses than cases with the wild-type BRAF.

BRAF mutation testing and targeted therapy has yet to be used in melanoma case management in Indonesia. These are due to limitations in both availability and costs. Currently, BRAF mutation testing and BRAF inhibitors are not included in the Indonesian national health insurance program. Healthcare centers that provide molecular mutation testing are also still scarce. Most melanoma patients in Indonesia cannot afford the price of completing a whole cycle of BRAF inhibitor therapy. As a result, *BRAF V600* mutation testing is only done in research settings and treatment for melanomas is commonly limited to surgery and cytotoxic chemotherapy (dacarbazine).

These difficulties suggest that currently it is only possible to provide molecular testing and treatment to a select portion of patients who would most likely benefit from it. The mutation rate could be a potential marker to predict response to targeted therapy. A study shows that higher *BRAF V600* mutation VAFs are associated with better response rates to vemurafenib [19]. Quantification of BRAF mutation levels may help select patients with the best potential for molecular treatment.

Another option would be to use a less sensitive method for clinical usage in Indonesia, such as RT-PCR. The facilities for this method are more widely available, easier, and cheaper in Indonesia than pyrosequencing. Although only tumors with higher VAFs would be detected by PCR studies, this would mean that the identified tumors will have better treatment response. Thus, using PCR methods for mutation detection may be more feasible and cost-effective for prognostic and therapy decision-making in Indonesia.

Our previous research provided the *BRAF V600* mutation prevalence in Indonesian skin nodular melanoma cases. This research improved it using a more sensitive method and specified the mutation subtype. Through these studies, we have advanced knowledge on *BRAF V600* mutation profiles in primary skin nodular melanoma cases in Indonesia. This information will help develop prognostication and treatment decision-making for Javanese patients.

## Conclusion

The *BRAF V600E* mutation was found in 53.85% of primary skin nodular melanomas, albeit with low variant allele frequencies. The high prevalence of mutations suggest that many Indonesian melanoma cases may benefit from molecular testing and targeted therapy, but more sensitive detection methods are needed for *BRAF V600* mutation status testing. However, availability and feasibility may need to be considered for usage in clinical practice. Further research is needed on the prognostic value of this mutation in Indonesian populations.

## Limitations

The small sample size was the main limitation in this study. Besides that, all the patients were Javanese people, the largest ethnic group in Indonesia. Data in this study might not be able to represent the entire Indonesian population which consists of more than 600 ethnic groups.

## Data Availability

All data generated or analyzed during this study are included in the submission. The raw data are available from the corresponding author on reasonable request.

## Abbreviations

DNA: Deoxyribonucleic acid
FFPE: Formalin-fixed paraffin-embedded
HRP: Horseradish peroxidase
MAPK: Mitogen-activated protein kinase
RT-PCR: Real-time polymerase chain reaction
TILs: Tumor-infiltrating lymphocytes
VAFs: Variant allele frequencies
